# Next-Generation Mars Network Position, Navigation, and Timing for Future Robotic and Human Explorers

**DOI:** 10.1007/s40295-026-00588-w

**Published:** 2026-06-08

**Authors:** Margaret Rybak, Todd Ely, Eric Gustafson

**Affiliations:** Jet Propulsion Laboratory, California Institute of Technology, Pasadena, CA USA

**Keywords:** Mars network, Constellations, Navigation, Global positioning

## Abstract

A next-generation Mars Network is investigated to determine a configuration optimized for both communications and positioning performance for surface users. A previously proposed 6000 km altitude, 3-satellite equatorial constellation that was found to be optimal for communications to surface users located in the latitude range from 60°S to 60°N is shown to be deficient for surface positioning. Inclining the 3-satellite configuration between 30° and 50° improves positioning performance to users in this latitude range; however, due to a lack of coverage this improvement is primarily seen for positioning when using tracking data collected over long timespans. Moving to an inclined 6-satellite case and using a Walker 6/2/0 delta configuration, at selected inclinations and altitudes, greatly improves the positioning solution performance over shorter timescales, with the best performance obtained with orbits inclined at 50°. Also examined were continuous coverage global constellations that were compared to the Walker 50°: 6/2/0 configurations. The single fold continuous coverage Walker 55.7°:7/7/5 constellation slightly improves the positioning performance and provides more uniform and continuous coverage to the poles, which the Walker 50°:6/2/0 case cannot. Finally, a Walker 57.1°: 8/8/2 constellation that provides continuous twofold coverage was examined; however, the high altitude required for this case reduces its communication performance and yields poorer positioning performance relative to the Walker 55.7°:7/7/5. It is concluded that a next generation Mars Network with focused support to users between 60°S and 60°N that the Walker 50°: 6/2/0 is the best positioning and communications performance while, for continuous coverage global coverage, the Walker 55.7°: 7/7/5 is superior.

## Introduction

The current Mars network is ad hoc consisting of Ultra High Frequency (UHF) relay services to surface users provided by orbiting science satellites (i.e., the Mars Reconnaissance Orbiter (MRO), Odyssey, Mars Atmosphere and Volatile EvolutioN orbiter, and the ExoMars Trace Gas orbiter that nominally use the Electra radio [1] for downlink commanding and uplink data relay. The network primarily supports relay passes that average 15 – 30 min in length and can occur several times a day. It also has a nascent capability to measure Doppler tracking, with first use demonstrated by Li, et al. [2], and recent results by Elliot, et al. [3] that assessed the ability to determine the global surface position of the InSight lander using Electra based Doppler data. They found < 10 m (3-σ) InSight position solution uncertainties using two-way Doppler and ~ 3 km (3-σ) using one-way Doppler using the current network capability. However, these data were collected over months with passes often separated by weeks. With the advent of human and increased robotic exploration of Mars in the coming decade, this level of performance will likely be insufficient and the need for an in-situ, GPS-like navigation service will be critical for safe, long-distance surface exploration. A dedicated constellation providing position, navigation, and timing (PNT) services (in conjunction with data relay services) could provide more *timely* and *accurate* global positioning to support this exploration. This research has focused on developing constellation designs and associated PNT services of a next-generation Mars Network (that we label Next-Gen MN) that could yield significantly more timely and accurate positioning performance then is available with the current network, while simultaneously minimizing the size and extent of the constellation.

We start our analysis with the Next-Gen MN constellation proposed by Gladden, et al. [4] that optimized for data throughput from a fixed surface user in the latitude range from 60°S to 60°N (which encompasses the likely range for human exploration and most robotic explorers from the past and current – NASA’s Phoenix lander is the exception that landed at 68°N latitude). Their analysis considered many factors and found that a small constellation consisting of three equatorial satellites at a 6000 km altitude in circular orbits was a “sweet spot” that provided near-constant network availability in the desired latitude range with sufficient data rate for the anticipated capability of higher-powered users. Furthermore, while each relay satellite communicated with users, they simultaneously had access to Earth roughly 90% of the time. However, they did not consider PNT services in their selection process. The focus of this research is on the PNT services/performance that such a constellation might provide and whether alternate architecture/constellations could improve PNT outcomes. Furthermore, given the cost to deploy spacecraft at Mars is high, any PNT satellite constellation should strive towards the smallest number of satellites that can provide reasonable services, ideally with combined communication and PNT services. This research is focused on the determining the smallest constellation that can provide “accurate” surface localization accuracies to users located, primarily in the latitude range from 60°S to 60°N, but globally as well. With a small constellation, the typical metric of finding an optimal geometric dilution of precision (GDOP) often used to evaluate terrestrial GNSS positioning, does not apply as instantaneous, soluble position solutions may not exist. Rather tracking data needs to be collected by a user over a period of time to be able realize a soluble position solution. A differentiating figure of merit for evaluating a PNT services adopted in this research is to determine a constellation configuration that yields accurate positioning in a minimal amount of time. Naturally, “accurate” and “minimal” may vary depending on user needs, in the present research we evaluate average positioning accuracy of users across the primary latitude range of interest as a function of time. The aim is to use these metrics for comparing constellation configurations to select the constellation with the best performing positioning outcomes with the smallest number of satellites and at the lowest altitude (to ensure desirable communication data rates/volumes are achievable). With this in mind, the Gladden constellation at 6000 km is a reasonable point of departure for this analysis and provides a measure with which, if the constellation altitude increases, the communication utility will naturally decrease.

A high fidelity PNT analysis has been developed using the Mission Analysis, Operations, and Navigation Toolkit Environment (Monte), JPL’s operational navigation software, that enables accurate assessment of the user PNT outcomes for a range of tracking and timing architectures [5]. These include:

1. One-way and two-way radiometric Doppler and range tracking,
2. Time division multiple access (TDMA) and code division multiple access (CDMA) modulation schemes,
3. Various user clock capabilities ranging from temperature controlled oscillators (TCXOs) to atomic clocks.

The analysis includes high fidelity modeling of dynamic and measurement errors affecting the PNT constellation ephemeris and timing accuracy and perform Monte Carlo simulations of selected scenarios. We collect sample statistics from Monte Carlo simulation of surface users located across Mars and assess their solution accuracies and compare them to their filter’s formal a posteriori uncertainties. This capability allows us to assess the value of a given constellation design/architecture for surface users in selected regions or globally (a later study will examine orbiting users of the Mars Network). By parametrically varying tracking data types and constellation orbit parameters, we can determine constellations that provide the best PNT outcomes for users.

## Simulation Setup

The simulation is a Monte Carlo with 20 realizations (i.e., a realization is a single run of the Monte Carlo) for all cases (one exception will be noted later) and lasting for a period of 4-days that is broken into two main segments. The first segment is for three days and focuses on estimation of the orbit and clock of each Mars Network spacecraft (MNSC) that make up the Next-Gen MN constellation using Earth-based tracking by the Deep Space Network (DSN). After reconstruction, the MNSC orbits and clocks are propagated over the fourth day to obtain their broadcast orbit and time ephemerides for use by the Mars surface user. The second period, beginning on the third day and lasting for a day, is for tracking by the Next-Gen MN to the Mars surface user and estimation of the user’s position, which have been placed in a range of latitudes depending on the expected Mars coverage by the network constellation.

Multiple constellation configurations were considered for tracking and positioning of the surface user, as outlined in Table 1. These configurations consider the tradeoffs between optimal communication and navigation performance, which will be discussed in depth in the results section. The first constellation is based on the 6000-km altitude, equatorial 3-satellite configuration proposed by Gladden, et al. [4]. As noted previously, this constellation maximized data throughput while also providing near-constant network availability. However, as we will show later, the equatorial satellites do not provide significant measurement diversity, especially near the equator, and is non-optimal for positioning. To provide more measurement diversity the Gladden 3-orbiter configuration was varied in inclination ranging from 10° to 60° (we’ll label these configurations as Gladden *i*: 3/3/0). Though this provides better positioning performance than the equatorial case, tracking gaps coupled with a lack of multi-fold coverage result in lengthy tracking periods required to attain reasonable performance. Thus, to explore constellations with higher density coverage at the mid latitudes, 6-satellite configurations at varying inclinations were investigated. Efficient constellations designs have been investigated for many decades, with a thorough survey of the many approaches recently documented by Choo, et. al. [6]. The most efficient single-fold global continuous coverage constellation using circular orbits is a 5-satellite Walker delta constellation [7, 8]. In recent years other constellation configurations, namely Flower constellations, have been analyzed; however, their application to PNT constellations have focused on larger ones (such as GPS or Galileo) [9, 10]. The general conclusion from these references is that it is possible for select designs to yield a smaller constellation than its Walker counterpart; however, it is typically a reduction of only 1 satellite from a set of over 24 (in the case of a fourfold coverage constellation). Because the constellations being considered in the present research are smaller, with at most twofold coverage, the Flower constellation design approach was not considered further. Inspired by the Walker delta configuration’s efficient packing of coverage areas, we have adopted this constellation configuration approach for the different designs that we investigated. Walker delta configurations are specified using four parameters that can be written in the form $i:T/P/F$ where $i$ is the satellite orbit inclination, $T$ is the total number of satellites, $P$ is the number of orbit planes, $F$ is the phasing factor used for phasing the satellite’s mean anomaly between adjacent planes. These parameters are used to configure the satellite orbits in the constellation using the following rules for right ascension of ascending node ${\Omega}_{j}$ of orbit plane $j\in \left[0,P-1\right]$ and the mean anomaly ${M}_{k}$ of satellite $k\in \left[0,\frac{T}{P}-1\right]$ using the following rules.

**Table 1:** Next-gen Mars Network Constellation Parameters Considered

Parameter	Gladden i: 3/3/0	Walker i: 6/2/0	onefold Global Walker 55.7°: 7/7/5	twofold Global Walker 57.1°: 8/8/2
$h=("i.e.," a=h+R)$	6000 km	6000 km	6511.8 km	17,984.0 km
$e$	0.0	0.0	0.0	0.0
$i$	$\left[0^\circ ,60^\circ \right]$	$\left[0^\circ ,60^\circ \right]$	55.7°	57.1°
$\Delta\Omega$	120°	180°	51.4°	45°
$\omega$	0°	0°	0°	0°
$\Delta {M}_{F},\Delta {M}_{T/P}$	0°, 0°	120°, 0°	0°, 257.1°	0°, 90°

1 $$\begin{aligned} & \Omega_j ={\Omega_0} +j{\Delta}{\Omega}=0^\circ +j\left( \frac{360^\circ}{P}\right)  \\ & M_k =M_0+j{\Delta}M_F+k{\Delta}M_{T/P}=0^\circ +j\left(F \frac{360^\circ}{T}\right)+k\left(360^\circ \frac{T}{P}\right) \end{aligned}$$

**Fig. 1:**
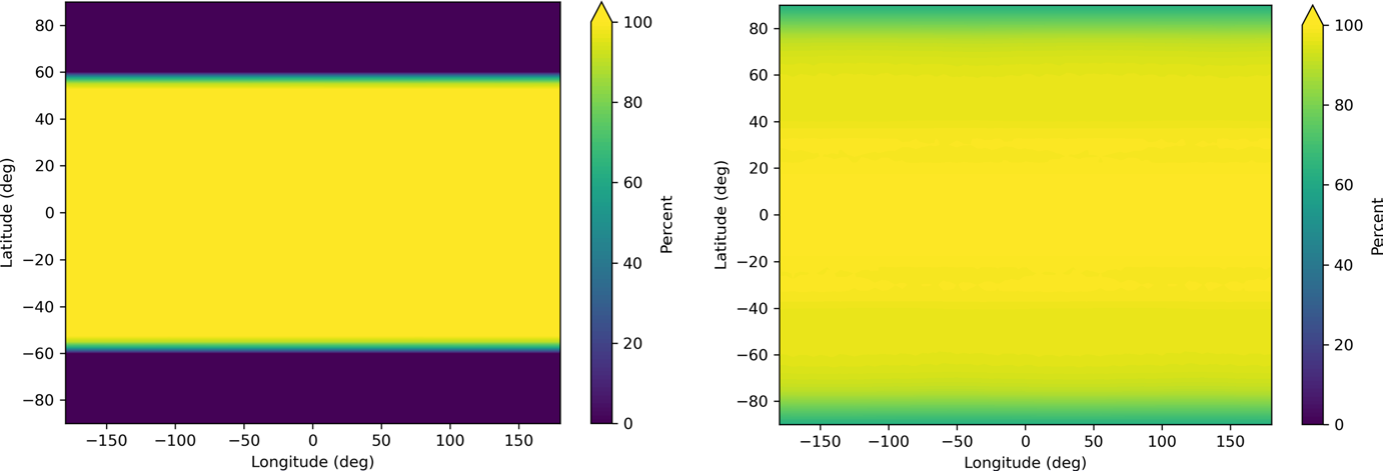
onefold percent coverage for the Walker 0°: 6/2/0 (left) and Walker 40°: 6/2/0 (right) constellations, both at a 6000 km altitude

All the orbits in a standard Walker delta configuration are circular, thus the eccentricity $e$ and argument of periapsis $\omega$ are both equal to zero. For computing the coverage of these orbiters, we have assumed a minimum elevation angle $\delta$ of 10° and, given the satellite’s orbit altitude $h$, the half central angle $\theta$ (or angular radius) of the coverage circle associated with the satellite conforms to

2 $$\mathrm{cos}\left(\theta +\delta \right)=\frac{{R}_{{\male}}}{{R}_{{\male}}+h}\mathrm{cos}\delta $$

where ${R}_{{\male}}=3396.19 km$ is the radius of Mars. As an example, $\theta =50.15^\circ$ for a 6000 km satellite with the 10° horizon mask and, for the Gladden 3 orbiter configuration (which has a Walker delta configuration of 0°: 3/3/0), the equator has ~ 84% coverage at any instance. Constraining the 6-satellite configurations examined to this altitude, to preserve communication data throughput, their inclinations ranged from $\left[0^\circ ,60^\circ \right]$ and, using the 6/2/0 Walker delta configuration, had a range of coverage properties. For example, the 0°: 6/2/0 configuration exhibited 100% onefold continuous coverage at latitudes between $\left[-55^\circ ,55^\circ \right]$ and the 40°: 6/2/0 configuration provided global onefold coverage over time that was still continuous at lower latitudes but had short tracking gaps near the poles (with a maximum of under an hour). These onefold coverage properties for the two constellation examples are illustrated in Fig. 1.

We also considered minimally sized onefold and twofold *continuous* global coverage constellations as determined by Walker [8], with the goal of comparing their performance to the *i*: 6/2/0 cases that do not provide continuous coverage at all locations. The onefold continuous coverage Walker constellation 55.7°: 7/7/5 with a 6511.8 km altitude (resulting from a required $\theta =60.3^\circ$) provides always in-view tracking to surface users located anywhere on Mars. Furthermore, this option requires only a modest increase in altitude relative to the *i*: 6/2/0 cases examined at 6000 km. Moving to twofold continuous coverage (enabling for kinematic 2-d positioning for latitude and longitude using two-way tracking), the smallest Walker configuration providing this level of coverage and with the lowest increase in altitude is the 57.1°: 8/8/2 constellation at a 17,984 km altitude ($\theta =71^\circ$). Its higher altitude (slightly more than areostationary) would significantly affect its data relay throughput capability but, if it were a dedicated PNT constellation which doesn’t require high data rates, it becomes a useful option for comparison to the lower altitude counterparts with a dual relay/PNT function.

### Tracking Network Modeling and Estimation

The network spacecraft dynamics are modeled using a 30 × 30 Mars spherical harmonics gravity field, gravity perturbations from the Sun, and n-body gravity from the solar system planets. Since the minimum altitude considered is at 6000 km, drag from Mars’ atmosphere is not active. A momentum desaturation also occurs in the middle of the orbit reconstruction period. A future study will consider the effects of non-gravitational acceleration from solar pressure mismodeling and/or outgassing would have on the broadcast orbit ephemeris error. A combination of two-way and one-way Earth-based tracking from the DSN is used to estimate and reconstruct the network satellite orbits and clocks over 3-days. Table 2 outlines the filter parameters used in the estimation with most being typical for Mars orbiters [11] and [12]. The types of parameters that comprise the orbit filter state include the following:

1. Dynamic parameters are for the position and velocity states of the orbiter and are related in time via the orbit equations of motion,
2. Random bias paraemters are estimated constants,
3. 1st-order Guass-Markov processes are an estimated stochastic parameter that are modeled with an exponential correlated Markov transition parameter,
4. Per pass stochastic parameters are random biases that change at the start of each tracking pass,
5. Consider parameters are random bias parameters that are not included in the filter’s state vector for estimation; however, their effect on the filter’s formal uncertainties are included via a senstivity matrix that maps the consider parameter uncertainties onto the estimated states.
6. Later, in the surface user position filter (Table 2) clock noise parameters are modeled using (Brownian motion) random walks for the phase and rate terms that are also correlated with each other. Model details can be found in Zucca [13].

**Table 2:** Tracking Network Spacecraft Filter Parameters

Parameter	Estimation Model	1-σ A Priori/Noise Strength
MNSC position	dynamic	10-km
MNSC velocity	dynamic	10 cm/s
Momentum Desaturation	bias	0.13 mm/s (= MRO desat requirement)
Mars Gm	consider	0.0014 km^3^/s^2^
Earth/Mars Ephemeris	consider	Full covariance
Earth Pole Motion	1st-order Gauss Markov	5-cm/Earth radius, 12-h batch, τ = 4-day
UT1	1st-order Gauss Markov	0.256/1000-s, 6-h batch, τ = 2-day
Troposphere	1st-order Gauss Markov	1-cm, 1-h batch, τ = 6-h
Ionosphere (day/night)	1st-order Gauss Markov	55-cm/15-cm, 1-h batch, τ = 6-h
Earth Station Locations	consider	Full covariance
2-way Range Bias	per pass stochastic	2-m @ start of each pass
Clock Bias	bias	0.1 μs
Clock Rate	bias	1 × 10^–13^ (DSAC retrace)

The stochastic parameters are updated in at their designated batch time which is the frequency with which the process noise is applied. The details of how Monte processes measurements and stochastic processes in a current state batch formulation are described in Ely [14].

A broadcast orbit and time ephemeris is then generated for a day following the reconstruction for each satellite. The spacecraft bus is assumed to be sufficiently sized to provide precision in-situ radiometric tracking and timing from a DSAC-class atomic clock to Mars surface users while simultaneously supporting high data rate transmission back to Earth using a 3-m high gain antenna (HGA), such as the one used by the Mars Reconnaisance Orbiter (MRO) that supports both X-band and Ka-band communications [11]. This constant connectivity to the Earth also enables constant two-way (and one-way uplink) tracking between the DSN and the network spacecraft, an assumption that becomes more practical when the DSN implements a Multiple Uplink Per Aperture (MUPA) capability that is currently being investigated [15].^2^

^2^ An alternate approach is via crosslinks connecting the spacecraft, which would maintain constant connectivity from surface users with the Earth through the crosslink connections and then on a single satellite link back to Earth. The crosslinks could also be used for radiometric tracking to augment the Earth link for orbit and satellite clock determination.

**Fig. 2:**
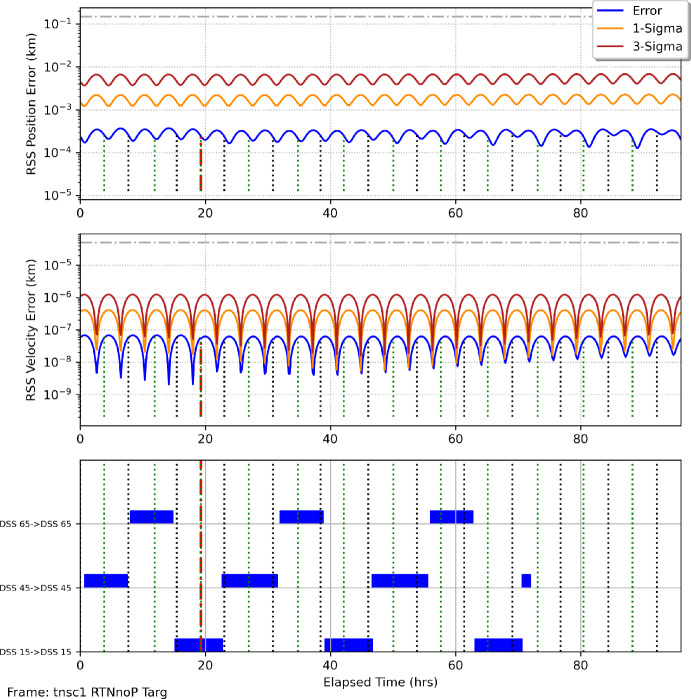
Gladden 50°: 3/3/0 typical MNSC position and velocity error and tracking showing the reconstruction over the first three days and predicts over the fourth day

**Fig. 3:**
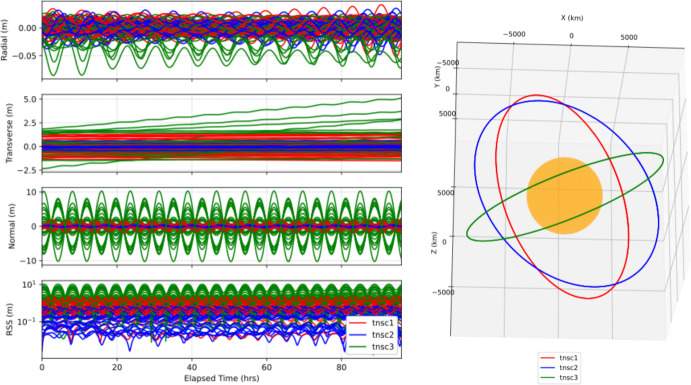
Gladden 50°: 3/3/0 MNSC smoothed position errors (left) with the x,y,z components representing radial, transverse, and normal coordinates, respectively. The first 3 days are reconstructed orbit errors and the 4th day are predicted orbit errors. 30 realizations of 3-orbiters in the constellation are shown. Corresponding orbit visualization (right) of the 3 orbiters around Mars. The color coding for each orbiter matches in the visualization and the error plots

**Fig. 4:**
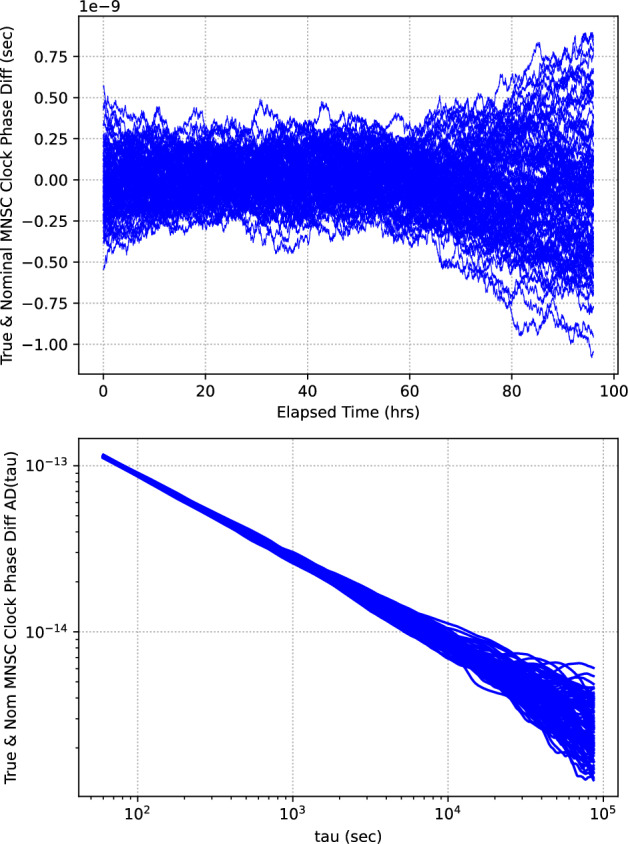
Twenty realizations of the Gladden 50°: 3/3/0 MNSC clock phase (top) with the first 3 days reconstruction and the 4th day is prediction and AD (bottom)

Figure 2 illustrates the typical Root Sum Square (RSS) position and velocity errors and 3-σ uncertainties after iteration and backward smoothing for one of the orbiters from the Gladden 50°: 3/3/0 constellation configuration and the associated DSN tracking during the three day reconstruction period and the behavior during the fourth day propagation period. A more statistical reprensentation of the reconstructed and broadcast orbit ephemeris errors from 20 realizations for simulated ‘truth’ of the same constellation (i.e., 90 trajectories in total) can be seen in Fig. 3. The smoothed spacecraft position errors are in a radial, transverse, and normal (RTN) components and their associated RSS, the figure shows that the error remains bounded below the 10 m level even during the propagation period over the fourth day. A corresponding 3D visualization of the three orbiters during the four day period is also shown. The camera view is along the vector pointing from the Earth to Mars in the EME2000 frame. This view informs the differences in position component errors. Two of the orbiters (colored blue and red in the figure) have a more face on geometry with respect to Earth in contrast to the third orbiter (green), which is more edge on. This results in larger errors overall for the third orbiter during this time period as there is less observability of the orbital components perpendicular to the Earth-based measurements. Each of the orbiters carries a DSAC-class of atomic clock (i.e., < 1.5 × 10^–13^ stability at 1 s and < 3 × 10^–15^ stability at a day) that enable providing precision in-situ radiometric tracking and timing services while, with DSAC’s low drift rates (orders of magnitude less than a typical rubidium space clock [16]), minimizing ground support requirements. If the orbiters carried clocks with lesser stability, the users would have to compensate for orbiter clock errors via estimating for them; thus, degrading their positioning performance when processing one-way range data. Figure 4 shows the DSAC phase time histories over the 4-day simulation and the associated Allan Deviations (AD) for the 20 realizations, which confirm DSAC-level of performance. Note the clock reconstruction is bounded by approximately ± 0.75 ns time error with this error growing during the propagation period. This level of performance is sufficient for the broadcast time ephemeris to users to be a simple linear fit of a time bias and rate.

### Surface User Modeling and Estimation

We investigate two tracking scenarios for the surface users; one is using two-way range and Doppler for determining the user’s position and the other is using one-way range and Doppler for the position estimation. One-way tracking utilizes CDMA modulation so that a user may track multiple satellites in-view simultaneously, while two-way uses TDMA modulation that permits duty cycling between a fixed number of in-view satellites (currently switching every 5 min between 2 satellites). The tracking network satellite broadcast orbit and time ephemeris is utilized by the surface user to obtain the MNSC location and time with the residual error in the ephemerides still present in the data. To compensate for the residual error in the orbit ephemeris, the user’s positioning filter state is augmented by estimating for the orbiter’s offset position errors with respect to its broadcast ephemeris using a white noise model with a noise strength of 9 m (3σ) (i.e., a bounding error as seen in Fig. 2 and Fig. 3). The users have a Miniature Atomic Clock (MAC) with the following properties [17]:

1. Stability of 3 × 10^–10^ 1-s, 1 × 10^–12^ at 1000 s, and a 1.7 × 10^–12^/day drift,
2. Dimensions of 5 cm × 5 cm × 0.7 cm,
3. Power use of 6 W at 25 °C.

**Fig. 5:**
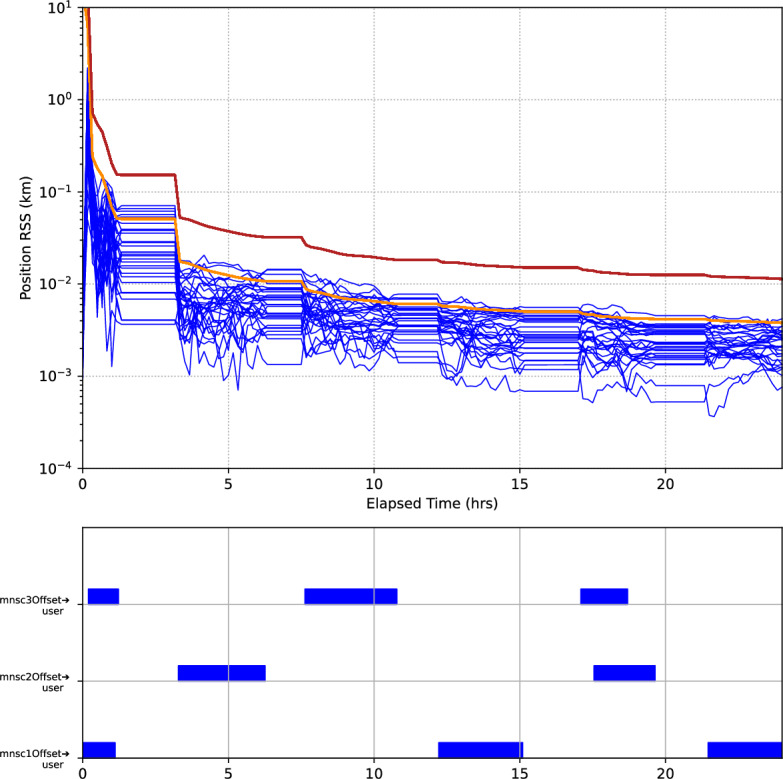
Position RSS and tracking passes for surface user at 30° latitude using one-way tracking from a Gladden 50°: 3/3/0 constellation

^3^ TCXOs were considered; however, since the tracking coverage is sporadic and typically not multi-fold, these oscillators were not adequate for yielding sufficiently accurate solutions using one-way tracking.

The MAC is amenable to smaller, lower powered users while still being significantly more stable than a typical TCXO.^3^ The user solves for their position and, if one-way range and Doppler, also their clock using a 2-d stochastic model developed by Zucca and Tavella [13]. Table 3 outlines the surface user filter parameters. Figure 5 shows 20 realizations of the position error RSS and tracking for a surface user at a latitude of 30°, using one-way tracking from Gladden 50°: 3/3/0 constellation. Note that the tracking passes (bottom of the figure) illustrate their variability for a given user with the coverage switching between twofold, onefold, or none over the course of the day. The implications of the performance for this constellation case will be discussed in the next section.

**Table 3:** Surface User Filter Parameters

Parameters	Estimate Type	1-σ A Priori/Noise Strength
User Position: lat/lon/radius	bias	(10-km/${R}_{{\male}}$, 10-km/${R}_{{\male}}$,10-km)
User Clock Bias Phase	bias	0.001 s (= typical initial value from telemetry-based time correlation)
User Clock Bias Rate	bias	5 × 10^–11^ s/sec (= MAC retrace)
User Clock Bias Acceleration	bias	10 × (1.7 × 10^–12^/day) (= 10 × MAC aging)
User Stochastic Clock Phase User Stochastic Clock Rate	correlated random walks (RWs)	Phase RW: 3 × 10^–11^ $\sqrt{s}$, Rate RW: 5.1 × 10^–14^ $1/\sqrt{s}$, 60 s batch
MNSC Offset Position Error Components	white noise	3 m, 60 s batch
2-way Range Bias	per pass	2-m @ start of each pass

## Surface user positioning performance for different tracking configurations

Various tracking constellation and surface user configurations were examined for both one-way and two-way PN range and Doppler tracking architectures. The latitude of the surface user was varied from 60°S to 60°N. The uncertainty was calculated for different tracking durations, from 1-h to 1-day. Later cases for the two continuous global coverage constellations extended the user latitudes to the poles.

### Gladden communication optimized 3-satellite equatorial configuration

**Fig. 6:**
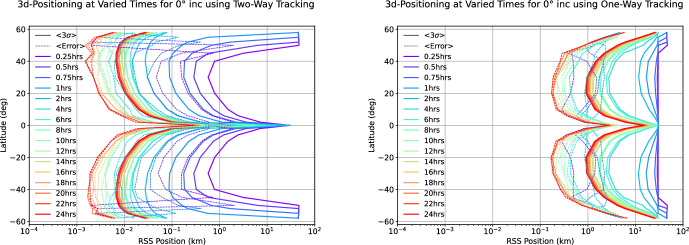
Three satellite equatorial constellation showing surface user 3d-positioning mean error and mean 3σ uncertainty at selected periods of tracking ranging from 1-h to 24-h using two-way (left) and one-way (right) tracking

The first orbit configuration analyzed was the 6000 km, equally spaced, equatorial, three satellite constellation proposed by Gladden, et al. [4] (i.e. the Gladden 0°: 3/3/0 case from Table 1) with a 20 realization per surface user Monte Carlo simulation. Figure 6 shows the surface user mean position error and mean 3σ uncertainty (where the means are of the 20 different realizations for each user) using two-way and one-way tracking from the three satellite orbiters. The latitude of the surface user is varied from 60°S to 60°N while the longitude is fixed to 135.62°E (the InSight lander’s longitude). The mean error and mean uncertainties are shown for different tracking durations, from 1-h to 1-day. The largest position uncertainty occurs for low latitude users due to poor orbital geometry. Indeed, at the equator the solution is degenerate, this stems from the fact that there is no North–South variation in the tracking passes by the orbiters. For example, at the InSight landing location (latitude of 4.5°) the position uncertainty is ~ 100 m (3σ), whereas with the current ad hoc Mars Network achieves about 10 m (3σ) using Electra based two-way Doppler [3]. This poor positioning performance is in contrast to the constellation’s communications performance that is optimal at the equator [4]. The solution improves as the user latitude increases, achieving position uncertainties after a day of tracking of < 10-m (3σ) using two-way tracking and < 1-km (3σ) using one-way tracking. This improvement tapers off at high latitudes due to the low elevation angle between the user and orbiter, which limits the tracking passes duration and geometric diversity. Note that the best performance is achieved in the mid-latitudes, for instance, after only an hour of tracking the position uncertainty is ~ 100 m (3σ) with two-way tracking.

### Gladden 3-satellite configuration, varied inclinations

**Fig. 7:**
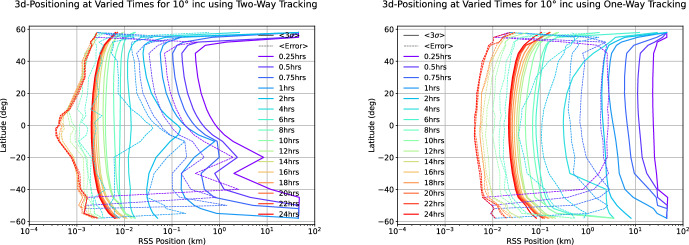
Three satellite 10° inclined, surface user error and 3σ uncertaintiy using two-way (left) and one-way (right) tracking

The poor PNT performance at the equator raises the question about other constellation orbits that would be better suited for positioning. To explore this, we varied the inclination of the three orbiters between 10° and 60° and found that position uncertainty significantly improves for all surface user latitudes when the orbiter inclinations are non-zero. Figure 7 shows the performance using 10° inclined orbiters for varied user latitudes and tracking durations. For two-way tracking, the uncertainties are greatly improved to < 10-m and 10 s of meters for the one-way case after a day.

**Fig. 8:**
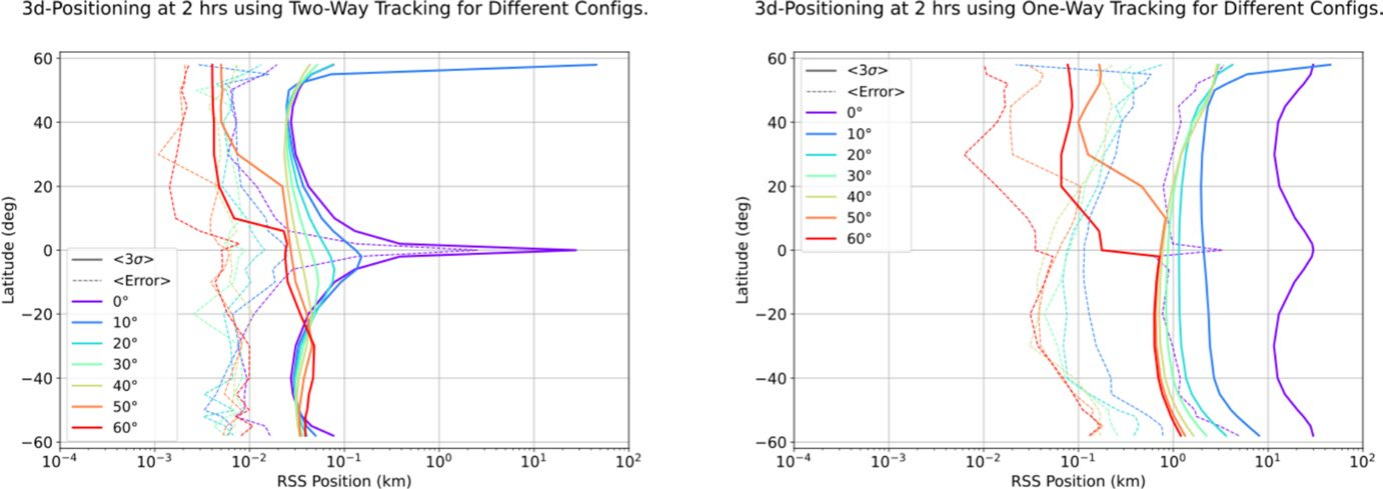
Three satellite constellations at various inclinations, surface user error and 3σ uncertaintiy using two-way (left) and one-way (right) tracking after 2-h

**Fig. 9:**
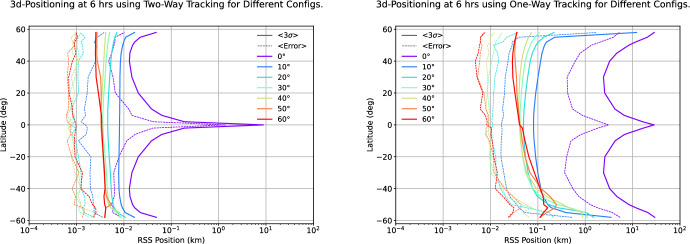
Three satellite constellations at various inclinations, surface user error and 3σ uncertaintiy using two-way (left) and one-way (right) tracking after 6-h

**Fig. 10:**
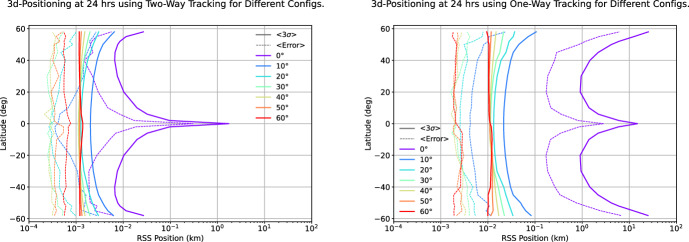
Three satellite at various inclinations, surface user error and 3σ uncertaintiy using two-way (left) and one-way (right) tracking after 24-h

Figure 8, Fig. 9, and Fig. 10 show the position uncertainties, when varying the orbit inclinations, after a duration of 2-h, 6-h, and 24-h respectively. Using orbiters inclined between 30° to 50° provide the best surface user performance. Uncertainties of < 1-m (3σ), and < 10-m (3σ) can be achieved for two and one-way tracking after 24-h, respectively. However, over shorter durations, there are configurations that occur when the view periods between the orbiter and user are limited, and result in windows of time where the improvement gained from the higher inclination is lost due to the limited tracking availability.

### Walker *i*: 6/2/0 satellite configurations

**Fig. 11:**
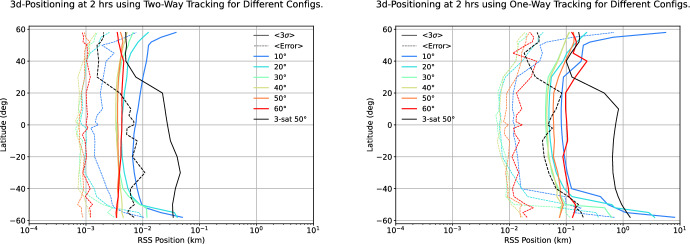
Walker *i*: 6/2/0 at various inclinations, surface user error and 3σ uncertaintiy using two-way (left) and one-way (right) tracking after 2-h

**Fig. 12:**
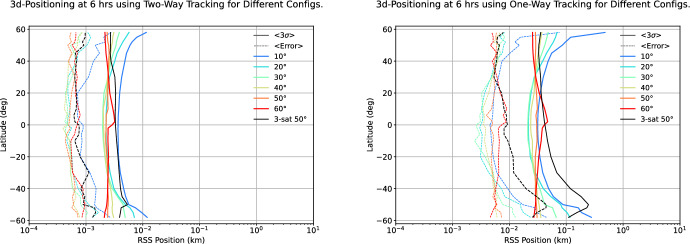
Walker *i*: 6/2/0 at various inclinations, surface user error and 3σ uncertaintiy using two-way (left) and one-way (right) tracking after 6-h

**Fig. 13:**
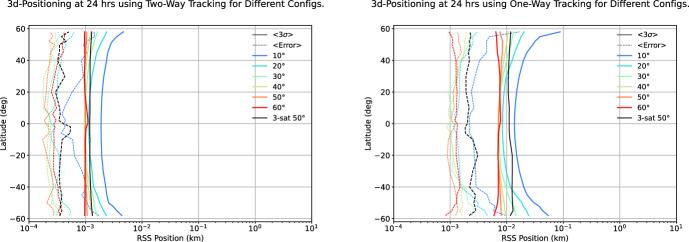
Walker *i*: 6/2/0 at various inclinations, surface user error and 3σ uncertaintiy using two-way (left) and one-way (right) tracking after 24-h

The next configurations investigated were the Walker *i*: 6/2/0 6000-km altitude satellite constellations at inclinations that varied from 10° to 60°. Recall, these configurations use the Walker delta formalism for specifying the orbit topology; however, they are not continuous global coverage constellations (which would require specified and fixed altitudes and inclinations, such as the 55.7°: 7/7/5 or 57.1°: 8/8/2 considered later). Instead, these constellations maintain the “sweet spot” altitude identified by Gladden [4], paired with the Walker configuration and tested at different inclinations, so that they can maintain useful dual use communications and PNT services. Figure 11, Fig. 12, and Fig. 13 show the surface user position uncertainties, at varied orbit inclinations, after durations of 2-h, 6-h, and 24-h. The performance for the Gladden 50°: 3/3/0 configuration is overlaid in black on the *i*: 6/2/0 results. After 2-h and for above 10° orbiter inclination, the user uncertainty is < 10-m (3σ) at all latitudes for two-way tracking and, restricting to latitudes between 40°S and 40°N, < 100 m (3σ) for one-way tracking. At 24 h and above 10° orbiter inclination, the positioning uncertainty achieves < 3-m (3σ) and about 30-m (3σ) in the full latitude range between 60°S and 60°N for two and one-way tracking after 24-h, respectively. These results suggest that the higher inclination constellations perform better with only small differences seen in the inclination range from 40°– 60°inclinations. This set also performs better than the Gladden 50°: 3/3/0 constellation at all times and latitudes.

### Coverage Differences between Gladden 50°: 3/3/0 and Walker *i*: 6/2/0 Constellations

**Fig. 14:**
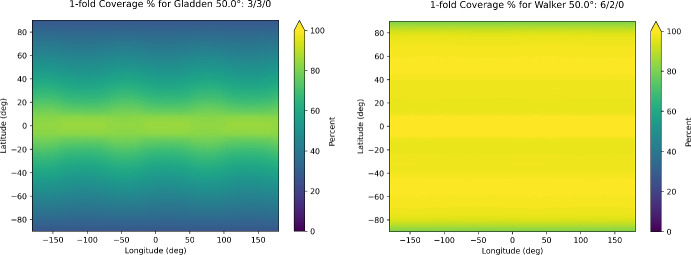
onefold percent coverage averaged over 8-days for the Gladden 50°: 3/3/0 (left) and Walker 50°: 6/2/0 (right) constellations

To better understand the performance over different timescales for the different constellation configurations, a coverage tool was developed. This tool finds the onefold and twofold percent coverage, mean gap, and mean pass from the constellation for a rectangular grid of users across the entire surface of Mars. Figure 14 shows the onefold percent coverage for the Gladden 50°: 3/3/0 and Walker *i*: 6/2/0 constellations, averaged over 8-days. As expected, the 3 satellite configuration results in overall less coverage than the 6 satellite one, with only the equatorial region getting near 100% coverage for the 3-satellite constellation. There is a narrow band of users near the equator that have similar onefold coverage between the Walker 50°: 6/2/0 and the Gladden 50°: 3/3/0 constellations and, examination of the positioning uncertainty seen near the equator in Fig. 12 after 6 h of tracking, shows that associated 3σ uncertainties differ by only a few meters for the two-way results. However, at the higher latitudes the Walker 50°: 6/2/0 constellation exhibits consistently higher levels of onefold coverage with overall being in the 80 to 100% range over the entire planet. Furthermore, when comparing the set of Walker *i*: 6/2/0 configurations, the Walker 50°: 6/2/0 results in the most consistent onefold coverage over the planet of this set.

**Fig. 15:**
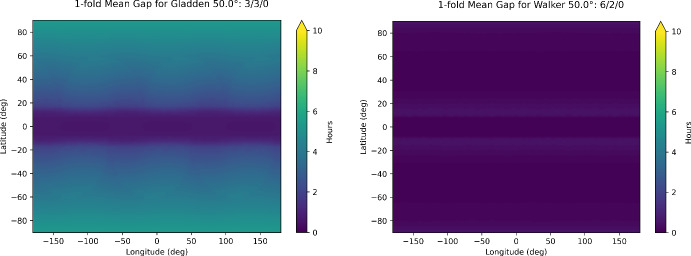
onefold mean gap averaged over 8-days for Gladden 50°: 3/3/0 (left) and Walker 50°: 6/2/0 (right) constellations

**Fig. 16:**
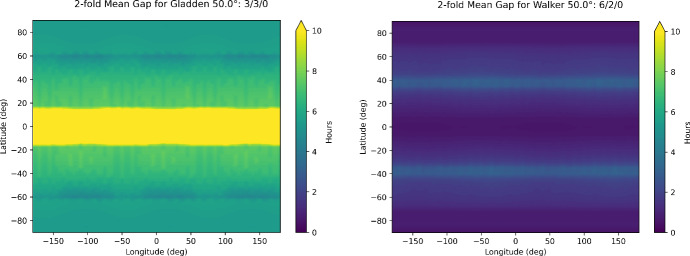
twofold mean gap averaged over 8-days for Gladden 50°: 3/3/0 (left) and Walker 50°: 6/2/0 (right) constellations

Figures 15 and 16 show the onefold and twofold coverage mean gap for both constellations. The Walker 50°: 6/2/0 has, not only minimal gaps for onefold coverage, but also a majority of twofold coverage for most surface user positions. This means better measurement geometry in addition to more tracking coverage overall. Two-fold coverage has a distinct advantage for positioning in that solution uncertainties converge much more quickly than with onefold. This twofold coverage advantage from the Walker 50°: 6/2/0 over the Gladden 50°: 3/3/0 is apparent with the 2-h 3σ uncertainties shown in Fig. 11, which are typically an order of magnitude less. For the two-way results at 2 h, the 6-satellite constellation achieves 2 m (3σ) uncertainties across all latitudes versus 30 – 40 m (3σ) at most latitudes with the 3 satellite constellation. For the one-way results at 2 h, the 6-satellite constellation achieves ~ 100 m (3σ) positioning at most latitudes, while the 3-satellite constellation positioning is on the order of a kilometer.

Given the superior positioning performance with the higher inclination Walker *i*: 6/2/0 and these onefold and twofold coverage considerations, the Walker 50°: 6/2/0 is the best performing constellation of the 3 and 6-satellite configurations considered.

### onefold Continuous Global Coverage Walker 55.7°: 7/7/5 Configuration

We turn our attention to constellations that explicitly provide continuous tracking and consider their performance relative to the best overall performing constellation so far, the Walker 50°: 6/2/0. First we examine the continuous onefold continuous global coverage using Walker 55.7°: 7/7/5 constellation at a 6511.8 km altitude. This altitude can be determined by obtaining coverage circle radius for a given onefold continuous coverage constellation as tabulated by Walker [8] and solving for the resulting altitude using Eq. (2) given the minimum elevation angle constraint adopted in this study (10˚). This constellation was selected because it is the smallest sized Walker constellation that remains close to the 6000 km altitude; hence, retaining the preferred data throughput capability while at the same time extending coverage to be continuous and global.

**Fig. 17:**
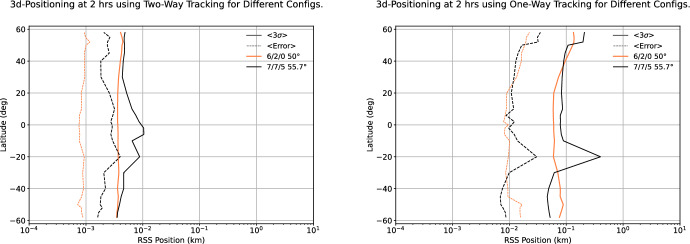
Positioning performance at 2-h for the continuous global coverage Walker 55.7°: 7/7/5 vs Walker 50°: 6/2/0 using two-way tracking (left) and one-way tracking (right) tracking

**Fig. 18:**
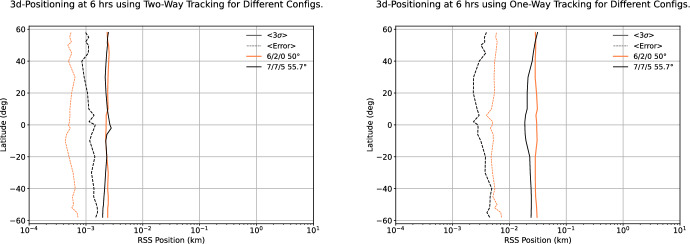
Positioning performance at 6-h for the continuous global coverage Walker 55.7°: 7/7/5 vs Walker 50°: 6/2/0 using two-way tracking (left) and one-way tracking (right) tracking

**Fig. 19:**
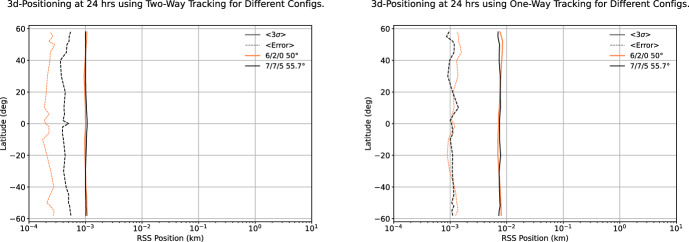
Positioning performance at 24-h for the continuous global coverage Walker 55.7°: 7/7/5 vs Walker 50°: 6/2/0 using two-way tracking (left) and one-way tracking (right) tracking

**Fig. 20:**
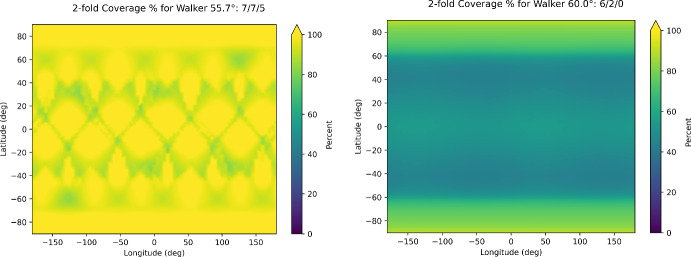
twofold coverage percent the Walker 55.7°: 7/7/5 (left) and twofold coverage percent for the Walker 50°: 6/2/0 (right)

Figure 17, Fig. 18, and Fig. 19 show the mean position errors and uncertainties between the Walker 50°: 6/2/0 and the Walker 55.7°: 7/7/5 after durations of 2-h, 6-h and 24-h for the two-way and one-way tracking scenarios focused on users in the latitude range from 60°S to 60°. For two-way tracking, the performance is slightly worse after 2-h in the mid-latitudes for the Walker 55.7°: 7/7/5 but still < 10 m (3σ), with performance converging to the 6-satellite constellation by 6-h and effectively the same at 24-h. The worse performance at 2-h could be attributed to the fact that the Walker 55.7°: 7/7/5 mean twofold gaps (averaged over 8 days) exhibit a greater longitudinal variation than seen with Walker 50°: 6/2/0. However, note that the performance variation is relatively small at 10 m (3σ) versus 5 m (3σ), and the Walker 55.7°: 7/7/5 constellation can provide more uniform coverage to the poles including continuous communications connectivity at any location, which the Walker 50°: 6/2/0 cannot. For one-way tracking at 2 h, the Walker 55.7°: 7/7/5 constellation shows better performance than the Walker 50°: 6/2/0 (with the exception of a few latitude locations, which might be statistical outliers) with largely 100-m (3σ) class uncertainties. The twofold percent coverage for Walker 55.7°: 7/7/5 and Walker 50°: 6/2/0 is shown in Fig. 20. It is conjectured that the presence of the 7th satellite results in a higher percentage of twofold coverage (as seen in Fig. 20) and higher n-fold coverage than with the 6-satellite configurations, resulting in better estimation performance for the user clock and better overall positioning. Indeed, at 6-h, the Walker 55.7°: 7/7/5 performs better at most latitudes than the Walker 50°: 6/2/0 but, by 24-h, the performance is largely the same between the two constellations and reaches < 10 m (3σ) across all the latitudes shown.

### twofold Continuous Global Coverage Walker 57.1°: 8/8/2 Configuration

We turn our attention to the Walker 57.1°: 8/8/2 constellation that provides continuous twofold global coverage. Unlike all of the constellations considered thus far, this constellation has to be at a much higher altitude, specifically 17,984.0 km, to achieve this level of coverage. This high of an altitude significantly reduces the constellation’s effectiveness as a communications relay because the average daily data throughput is reduced by over 6 times relative to one at 6000 km [4]. However, if we consider the constellation to be dedicated to only providing PNT services (and not data relay), then it can operate at lower data rates (since both the broadcast orbit and clock and PN ranging can be designed for lower data rates) and could likely use a smaller, less expensive spacecraft platform than would be needed for a dual purpose data relay and PNT constellation. Given this it is worth examining the relative positioning performance of this configuration to the others to determine if there would be benefit to a modestly sized dedicated PNT system. Besides altitude, another noteworthy difference with this constellation is that the orbit determination of the satellites is worse than with its lower altitude counterparts. Recall that for the 6000 km altitude orbiters the average orbiter RSS position uncertainty was < 10 m (3σ), whereas for the Walker 57.1°: 8/8/2 configuration the uncertainty varied typically between 40–50 m (3σ) with some orientations reaching 100 m (3σ). As a consequence, the surface user’s positioning filter compensates for this by increasing the MNSC offset position error component noise strength to 15 m (1σ) (from 3 m).

**Fig. 21:**
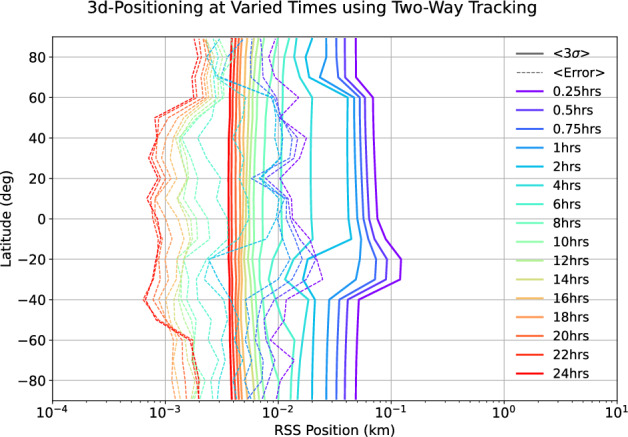
Continuous twofold coverage Walker 57.1°: 8/8/2 constellation showing surface user 3d-positioning mean error and mean 3σ uncertainty at selected periods of tracking ranging from 1-h to 24-h using two-way tracking

The 3d-positioning performance obtained from this constellation for users located at latitudes between 89°S to 89°N (i.e., global) using two-way tracking at times ranging from 15 min to 24 h are shown in Fig. 21. With only 15 min of tracking time, most users obtain < 100 m positioning uncertainty. The variations seen at 20°S and 30°S are likely due to the temporal nature of the coverage *greater than* twofold for the constellation, which will vary based on the epoch and initial location of the orbiting satellites at this epoch. That is, at different times and different locations, than seen in Fig. 21, may exceed the100 m (3σ) uncertainty level but the other locations would still be < 100 m (3σ). As the tracking period increases, these variations diminish and the performance becomes more uniform across all latitudes, which is achieved between 6 – 8 h for this constellation and reaches ~ 10 m (3σ). By 24 h, all latitudes have ~ 5 m (3σ) position uncertainty. It is noteworthy that, even though there are more spacecraft in total and more in-view because of their higher altitude, the lower altitude constellations examined achieve smaller overall position uncertainties. For instance, after 24 h of tracking the Walker 55.7°: 7/7/5 achieves ~ 1 m (3σ) position uncertainties across all latitudes. This is because, relative to the lower altitude counterparts, the orbiter ephemeris errors for the Walker 57.1°: 8/8/2 are larger, plus the orbiters move more slowly across the sky resulting in less geometry change for a given amount of time and, consequentially, less information content in the set of data used for user position estimation.

**Fig. 22:**
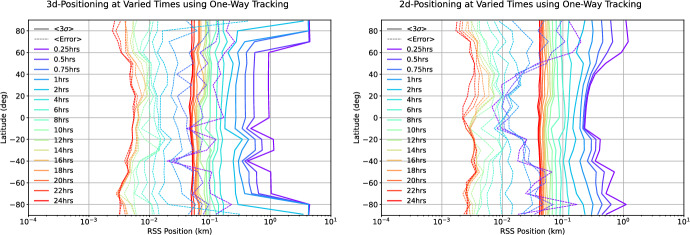
Continuous twofold coverage Walker 57.1°: 8/8/2 constellation showing surface user 3d-positioning (left) and 2d-positioning (right) for the mean error and mean 3σ uncertainty at selected periods of tracking ranging from 15-min to 24-h using two-way tracking

Turning to one-way tracking, positioning performance for 3d positioning and 2d positioning (i.e. latitude and longitude only) are shown in Fig. 22. As is the case with the prior constellations, the one-way performance is worse because the user must solve for their clock in addition to their position and the orbiter error. One method to reduce the impact of the extra clock estimation states is to reduce the position estimation to be 2 dimensional and only include latitude and longitude (where altitude can be determined by referencing an associated digital elevation map). Comparing the 3d results (left in the figure) and the 2d results (right in the figure), it is clear that the 2d-positioning performs significantly better than 3d. Within 15 min of tracking, the 2d-positioning yields 1 km (3σ) or less uncertainties across all latitudes. At 2 h, the 3σ 3d-position uncertainty is ~ 300 m (except at the poles) and the 2d-position uncertainty is ~ 200 m, at all latitudes. By 24 h, the 3σ 3d-position uncertainty is ~ 50 m and the 2d-position uncertainty is ~ 40 m, at all latitudes. Recall that the a priori RSS uncertainty is 52 km (3σ). If the a priori uncertainty were less (a likely scenario), then the 15 min positioning results would also likely be less than the 1 km levels seen here and, for the longer tracking times, relatively less uncertainties than are shown in Fig. 22 (left) – quantifying this is an area of future work.

### Next-Gen Mars Network Constellation Recommendation

Given that the Walker 55.7°: 7/7/5 positioning performance is measurably better than the Walker 57.1°: 8/8/2 and it is able to be an effective communications constellation, we find that the Walker 55.7°: 7/7/5 is a preferable configuration for global support and, if support is limited to the 60°S to 60°N latitude range, the Walker 50°: 6/2/0 might be preferred because of its smaller size.

## Conclusion

This research focused on the investigation of different constellation configurations for a Next-Gen MN. It was shown that the Gladden proposed equatorial 3/3/0 constellation, which maximizes data throughput and provides near-constant network availability, is deficient for positioning due to poor measurement geometry. Inclining the orbits of this constellation improves the positioning performance. However, the improvement is primarily seen for long timespans (24-h) due to limited coverage. Using an inclined Walker 6/2/0 configuration significantly improves the coverage and allows for markedly better solutions over shorter timespans, with the best performance occurring using an inclination of 50°. The use of a Walker 55.7°: 7/7/5 constellation that provides continuous global onefold coverage, enabling for continuous communication to a user located anywhere on the globe, yields positioning performance that is similar to the Walker 50°: 6/2/0 in the mid-latitudes but also extends it to the poles.

Future work would extend the users to be both orbiting and Mars approaching. The analysis also assumed near constant Earth-based tracking of the satellites (needed for continuous connectivity to Earth); however, analyzing a constellation with crosslinks and a single continuous ‘trunk’ line to Earth is an architecture that should be investigated. Finally, the use of orbit pairs for the TDMA measurements were chosen at random. These pairings could instead be optimized to provide the best measurement content and geometry.

## Data Availability

All datasets used during the current study were simulated as described in the manuscript.
